# Proceso educativo en biohuertos de plantas medicinales en personal de un establecimiento de salud peruano

**DOI:** 10.1016/j.aprim.2023.102598

**Published:** 2023-03-17

**Authors:** Mercedes Acosta-Román, Charles Frank Saldaña-Chafloque, Dionisio Ignacio Poma-Poma

**Affiliations:** a Universidad Nacional Autónoma de Tayacaja Daniel Hernández Morillo, Tayacaja, Huancavelica, Perú; b Hospital Regional Docente Clínico Quirúrgico Daniel Alcides Carrión, Huancayo, Perú

En el mundo la atención de patologías crónicas y su costo influyen para que los pacientes busquen profesionales en medicina tradicional y complementaria (MTC). Siendo la demanda mayor en usuarios de África, Asia, Australia y América del Norte. Este servicio de MTC está basado en algunos casos en tratamientos y procedimientos con hierbas medicinales^1^. En los países en vías de desarrollo de Latinoamérica, el sistema de salud no está aplicado íntegramente al enfoque intercultural, aunque más de 90% de las personas usen plantas medicinales dadas de su experiencia ancestral, lo cual se evidencia al no estar inmersas en sus políticas de salud, las barreras de los sistemas de salud y por parte del equipo de salud^2^. Actualmente, los profesionales de salud de algunos establecimientos de la sierra del Perú toman en cuenta los aspectos de interculturalidad en las atenciones que realizan^3^, ^4^. Asimismo, la interculturalidad en salud es un aporte en la atención del personal de salud integrando el aspecto cultural, atendiendo a los pobladores y respetando sus patrones culturales^5^. En 2021, al aplicar un instrumento de diagnóstico a 24 trabajadores de la microrred de salud del distrito de Colcabamba, que pertenece a la provincia de Tayacaja, del departamento de Huancavelica del Perú, donde 100% se encuentran dispuestos a recibir capacitaciones en temas de biohuertos de plantas medicinales, 64% no conocía las propiedades de estas, 91% no conocía sobre su preparación, 27% en algún momento ha indicado al paciente que utilice plantas medicinales y 100% cree que sería importante implementar centros de consejería para el uso de plantas medicinales. Por lo antes descrito, el objetivo de esta investigación fue conocer lo aprendido de un proceso educativo en biohuertos de plantas medicinales dirigido al personal de la microrred de salud Colcabamba, una experiencia de investigación cualitativa, de diseño de acción participativa sobre educación en salud en el contexto de la COVID-19. Con el muestreo no probabilístico por conveniencia, se trabajó con 33 participantes, entre médicos, enfermeros, obstetras, odontólogos y técnicos en enfermería; se realizaron cuatro capacitaciones: biohuerto de plantas medicinales, uso de hierbas medicinales, uso de arbustos-árboles medicinales y la cuarta en tipos de preparados. Las capacitaciones aplicando la modalidad de grupo de WhatsApp, con el envío del material digitalizado y audios de exposición, para reforzar con un seguimiento a base de llamadas a todos los participantes y así resolver sus dudas. Los resultados obtenidos fueron satisfactorios al culminar el proceso educativo, en las pruebas de calificación vigesimal, se halló en la prueba de entrada que 82% resultaron desaprobados con notas entre 7 y 10, el resto con 18% aprobados con notas de 11 y 12; todo lo contrario en la prueba de salida, 100% aprobados, 55% con notas de 17 y 18, 45% con notas de 19 y 20. Incluso los participantes al calificar las capacitaciones en su encuesta de satisfacción mencionan que de forma general fue excelente en 42% y bueno en 51%. Se concluye que lo aprendido servirá para la mejora en el manejo del biohuerto de plantas medicinales, que tiene como proyecto el establecimiento de salud y el conocimiento adquirido sobre el manejo terapéutico de plantas con propiedades medicinales, que replicarán en la atención de los pacientes, pudiendo así ofrecer una atención que respete la interculturalidad en salud.

## Financiación

Este trabajo no ha recibido ningún tipo de financiación.

## Conflicto de intereses

Los autores declaran no tener ningún conflicto de intereses.
